# The Dysfunctional Mechanisms Throwing Tics: Structural and Functional Changes in Tourette Syndrome

**DOI:** 10.3390/bs13080668

**Published:** 2023-08-10

**Authors:** Jacopo Lamanna, Mattia Ferro, Sara Spadini, Gabriella Racchetti, Antonio Malgaroli

**Affiliations:** 1 Center for Behavioral Neuroscience and Communication (BNC), Vita-Salute San Raffaele University, 20132 Milan, Italy; 2 Faculty of Psychology, Vita-Salute San Raffaele University, 20132 Milan, Italy; 3 Department of Psychology, Sigmund Freud University, 20143 Milan, Italy; 4 Division of Neuroscience, Scientific Institute Ospedale San Raffaele, 20132 Milan, Italy

**Keywords:** Tourette Syndrome, tic, motor control, neural plasticity, basal ganglia, striatum, dopamine, endocannabinoids, motor cortex

## Abstract

Tourette Syndrome (TS) is a high-incidence multifactorial neuropsychiatric disorder characterized by motor and vocal tics co-occurring with several diverse comorbidities, including obsessive-compulsive disorder and attention-deficit hyperactivity disorder. The origin of TS is multifactorial, with strong genetic, perinatal, and immunological influences. Although almost all neurotransmettitorial systems have been implicated in TS pathophysiology, a comprehensive neurophysiological model explaining the dynamics of expression and inhibition of tics is still lacking. The genesis and maintenance of motor and non-motor aspects of TS are thought to arise from functional and/or structural modifications of the basal ganglia and related circuitry. This complex wiring involves several cortical and subcortical structures whose concerted activity controls the selection of the most appropriate reflexive and habitual motor, cognitive and emotional actions. Importantly, striatal circuits exhibit bidirectional forms of synaptic plasticity that differ in many respects from hippocampal and neocortical plasticity, including sensitivity to metaplastic molecules such as dopamine. Here, we review the available evidence about structural and functional anomalies in neural circuits which have been found in TS patients. Finally, considering what is known in the field of striatal plasticity, we discuss the role of exuberant plasticity in TS, including the prospect of future pharmacological and neuromodulation avenues.

## 1. Introduction

Originally described more than a century ago, Tourette Syndrome (TS) is a neurodevelopmental disorder with a spectrum of neurological, behavioral, and cognitive characteristics consisting of multiple motor tics and including, not necessarily simultaneously, at least one vocal/phonic tic, lasting for a minimum period of one year [1]. It usually appears in childhood and can persist into adulthood [2]. It is recognized as a common neuropsychiatric disorder, with a privileged position between neurology and psychiatry. TS has an incidence of about 1% [3] and implies a considerable social stigma and a lower quality of life. Many studies show that this syndrome is a heterogeneous and non-unitary condition, as suggested by the diagnostic criteria of the American Psychiatric Association (DSM-5) and those of the World Health Organization (ICD-10). Thus, one type or phenotype of TS consists of “pure simple motor and vocal tics only”, while other clinical presentations include complex tics and comorbid disorders, various coexisting psychopathologies, and complex behaviors [4]. More precisely, the diagnostic criteria in DSM-5 are the following: “A. both multiple motor and one or more vocal tics have been present at some time during the illness, although not necessarily concurrently. B The tics may wax and wane in frequency but have persisted for more than 1 year since first tic onset. C. Onset is before age 18 years. D. The disturbance is not attributable to the physiological effects of a substance (e.g., cocaine) or another medical condition (e.g., Huntington’s disease, postviral encephalitis)” [5]. Concerning comorbidity, the most commonly observed concomitant neuropsychiatric disorders in TS include: attention deficit hyperactivity disorder (ADHD), obsessive-compulsive disorder (OCD), and depression. Comorbidity occurs in about 90% of these patients and only about 10% have “Tourette Syndrome alone” with no other psychiatric diagnoses [4]. Regarding the relationship between TS and ADHD, in approximately 60–80% of TS patients, this comorbidity exists [6]. Though, in TS and OCD cases, the percentage varies from 27 to 32% [2], even if several studies have shown that the obsessive-compulsive symptoms in TS are significantly different from the symptoms found in the “pure” or “primary” OCD. Robertson argues that the prevalence of depression among TS ranges from 1.8% to 8.9%, with a lifetime risk of 10% [7]. The relapsing and remitting course has been associated with significant psychopathology, including emotional lability, night fears, bedtime rituals, cognitive deficits, separation anxiety, oppositional behaviors, and hyperactivity.

In this review, after introducing the main manifestations and etiopathological theories of the syndrome, we will summarize and discuss the available evidence about the structural and functional anomalies at neural circuits which have been involved in TS, provided by studies on human patients. Finally, based on well-established neurophysiological knowledge, we suggest a possible hypothesis about the role of striatal synaptic plasticity in tics expression.

## 2. Symptomatology and Etiopathology of Tourette Syndrome

In TS, tics are sudden, repetitive, rapid, non-rhythmic, and stereotyped movements or language productions involving discrete muscle groups [8]. They typically occur several times a day or intermittently with an increasing and decreasing course, and their anatomical location, number, frequency, complexity, type, and severity usually change over time. Although the age at onset is 6–7 years, phonic tics usually arise later, at around 11 years [9], and the peak of severity is at around 10–12 years [10]. Tics can be simple or complex and are usually voluntarily suppressed [10]. It is possible to divide TS tics into motor and phonic ones. Simple motor tics are sudden, short, and meaningless movements, usually lasting less than 1 s, such as blinking, grimacing and mouth movements, and pouting lips. Motor tics usually begin in the head and face in early childhood with transient attacks of simple motor tics (such as blinking, the most common one). Over time there may be a “rostral-caudal” progression, i.e., from head and neck to shoulders, arms, and torso [8], but this trend cannot be predicted. Paroxysms are observed in a large number of patients and can be defined as continuous orchestrated manifestations of simple and complex motor tics [10]. Phonic tics begin 1 to 2 years after the onset of motor ones and can be divided into simple tics (coughing, guttural noises, nose marks, wheezing and whistling, and popping of the throat), complex tics (recitation of the last word or phrase heard, i.e., palilalia and echolalia, use of obscene expressions, i.e., coprolalia, but also murmurs, whistles and stereotyped verbalizations), “ideal” tics (a combination of motor and vocal tics) or “compulsive” tics. In the most severely affected patients, motor and phonic tics are often accompanied by various forms of complex tics [11]. Copropraxia can be defined as the execution of obscene gestures and is reported in 3–21% of patients with TS, while ecopheonomena occurs in 11–44% of patients [9]. These complex motor tics are rarely observed in the absence of simple motor tics [8]. Most patients with TS report some discomfort or a feeling of pressure before a tic called a “warning impulse”, which decreases during goal-directed behavior and increases with emotional arousal and fatigue [7]. TS adults and adolescents refer to phenomena that cause tics as “premonitory sensory impulse” or “premonitory urge.” A feeling of relief often accompanies the successful execution of a tic [12]. The pulse usually arises more than three years after the onset of tics.

TS etiology is very complex, with strong genetic influences, repeated streptococcal infections, and also pre and perinatal phenomena [13]. There is accumulating evidence that immune dysregulation contributes to the pathophysiology of OCD, TS, and Pediatric Autoimmune Neuropsychiatric Disorders Associated with Streptococcal Infections (PANDAS). TS patients may, in fact, have a predisposition to autoimmune responses or impaired general immunity; recently, beta-hemolytic streptococcal infections and/or an increase in anti-basal ganglia antibodies have been found in patients with TS [4]. For these reasons, Robertson suggests that diagnostic criteria should include: the presence of OCD and/or tics, the onset of prepubertal symptoms, association with group A beta-hemolytic streptococcal (GABHS) infections, episodic course of symptom severity, and association with neurologic abnormalities. It should be emphasized that PANDAS and TS are not the same entity. A study [14] investigated the potential role of microglial dysregulation in these clinical syndromes since microglia, in neurodegenerative diseases, is classically associated with inflammation, neural damage, and neurodegeneration [15]. It is established that the execution of most voluntary movements is entrusted to the paths originating from the primary motor area (pyramidal system), while the control of motor program execution is processed by areas connected to the extrapyramidal system [16]. Compared to patients with other types of hyperkinesia, patients with TS consider their dysfunctional movements as largely voluntary [17]: such an aspect suggests that both pyramidal and extrapyramidal motor systems are likely implicated in the syndrome. Importantly, blood flow and metabolism studies assessed the implication of dopaminergic transmission in the pathophysiology of TS, especially in or around portions of the caudate nucleus of the ventral striatum, a key region for motor and behavioral expression [18]. In fact, this syndrome is characterized by an excess of dopamine or hypersensitivity of the postsynaptic dopaminergic receptors at the BG level, with a consequent imbalance of the cortico-striatum-thalamus-cortical circuits [16].

## 3. Evidence from Animal Models of Tourette Syndrome

A plethora of animal models are able to mimic tic-like behaviors observed in patients suffering from TS. Most of them have been developed in rodents (for extended reviews, see [19,20,21]), but also primate models exist [22], including a recent one showing vocal tics [23]. Most of the developed models are based on the hypothesis of neurotransmitter systems dysregulation, which has been evidenced in TS patients, as well as in functional disturbance of the BG, as shown above. For this reason, most models make use of pharmacological manipulation of these systems, either systemic or localized at BG structures, such as the striatum. Clearly, such approaches provide behavioral evidence in the form of tics or anomalous stereotyped motor and non-motor behaviors, further supporting the starting hypotheses. In addition, if the modulation of the neurotransmitter system is localized and phenotypically specific, this provides more precise evidence about the classes of neurons and subregions involved in the generation of tics. Seminal examples of these studies include those based on striatal disinhibition using GABA blockers, which produced motor tics in primates [24] associated with changes in the electrical activity of motor cortex and BG nuclei, including peri-tic increase, decrease, or multiphasic variation of spike activity at distributed neurons of the globus pallidus externus (GPe) and internus (GPi) [24]. The study by Worbe and colleagues exploited injections of bicuculline (a GABA_A_ antagonist) at different sites of the BG, which were later traced using immunostaining so that specific anomalous motor behaviors could be related to the disinhibited regions [25]. Somatotopic organization of tics was observed with higher reliability in the rat version of the TS model based on striatal bicuculline injection [26]: rostrocaudal direction of tics, from the head to the forelimb and then to the hindlimb, was observed by injections sites in the anteroposterior direction [26]. In another study adopting striatal disinhibition in rats, the timing of tic expression as well as single-unit neuronal activity in the striatum, were found to depend on the electrical activity of cortico-striatal excitatory afferents [27]. The use of GABAergic antagonist picrotoxin in the dorsal striatum of mice was effective as well, and this model was used to show that cortico-striatal glutamatergic transmission is required for the expression of tics [28]. More recently, a study by McCairn and colleagues (2016) has shown, for the first time, that disinhibition in the ventral striatum of monkeys by monolateral injection of bicuculline in the nucleus accumbens (NAc) can elicit specifically vocal tics [23]. Interestingly, these tics were evidenced to involve the activation of a vast cortico-limbic network, including the anterior cingulate cortex (ACC), the amygdala, and the hippocampus, and peculiar electrophysiological correlates with local field potentials oscillations displaying higher coupling in the alpha band between NAc, ACC, and M1 [23]. Alternative strategies included the use of dopamine receptor antagonists [29] or pharmacological lesions of the nigro-striatal pathway [30], which better clarified the involvement of specific receptors (D1 and D2) and their interactions, as well as the ameliorating effects of commonly used medications for TS. In particular, co-administration of haloperidol (i.e., D2 antagonist and one available treatment for TS) together with D1 stimulation in rodents prevented the induction of complex behavioral super-stereotypy [29]; moreover, by chronically modifying the balance between direct and indirect pathways in juvenile rats that underwent striatal lesion, movements resembling tics were induced after quinpirole (i.e., D2/D3 receptor agonist) administration and associated to altered D1 and D2 RNA expression in post-mortem analyses [30]. Thus, also transgenic models contributed to investigating the neurobiological bases of TS. In the study by Nordstrom and Burton (2002), D1CT-7 transgenic mice, a very valuable model of TS with high face, construct, and predictive validity [31], were found to exhibit OCD-like compulsions and TS-like tics [32]. The authors suggest that the TS+OCD phenotype of D1CT-7 mice relates to the anomalous activation of glutamatergic projections to the striatum which has been shown in these animals [33]. In addition, dopamine transporter knockout was also effective for generating complex sequential stereotypy in rodents, albeit the neurophysiological correlates have not been addressed [34]. Recently, chemogenetic manipulation of dopamine receptor-containing neurons in the BG of a pharmacological mouse model of TS has been used to show that tic-like behaviors can be ameliorated by the inhibition of D1/D2 receptor-containing neurons in the substantia nigra and in the dorsal striatum, suggesting a specific role of these cells in the expression of TS symptomatology [35]. Another study exploiting chemogenetic manipulation addressed a rare genetic cause of TS using a mouse which is a knockout for histidine decarboxylase (Hdc) [36]. The authors found specific involvement of the histamine 3 receptor (H3R) in the production of stereotypies in these mice and that the activity of dorsal striatum neurons is required and sufficient to produce these motor symptoms [36]. Unfortunately, in all the studies referenced above, direct perturbation of a neurotransmitter system was adopted: this condition can lead to complex interaction effects that might limit the significance of the results and their relevance to TS development. Interestingly, more advanced approaches based on optogenetics have implicated both the activation of the cortico-striatal pathway [37] and the nigro-striatal pathway [38] in the generation of tics, supporting the results described above but without the use of any other tic-inducing manipulation. Furthermore, using an optogenetic approach, Burguière and colleagues were able to show that the pathway connecting the orbitofrontal cortex to the dorsal striatum can suppress compulsory motor activity observed in a transgenic model with deletion of the synaptic scaffolding gene Sapap3 [39]. On the other hand, models based on other etiopathological factors [19], including immunological ones [20], can provide more reliable observations on the spontaneous neurobiological alterations that might play a role in TS. Unfortunately, most of these studies focused only on the behavioral outcome or on the localization of immunological targets, rather than on neurophysiological analyses. Nevertheless, due to the relevance of their results to the context of synaptic plasticity, studies based on immunological manipulation will be discussed later in this review. Starting from omics data, some findings may be of interest in the definition of TS etiopathogenesis. For example it was seen, by RNA sequencing, that there was a decrease in the number of GABA-ergic and cholinergic interneurons as well as an activation of microglia in caudate and putamen of TS patients [40]. Similarly it was observed [41] that the FLT3 (Fms Related Tyrosine Kinase 3) gene, encoding for a tyrosine-protein kinase associated with immune and inflammatory functions, was found overexpressed at the level of the dorsolateral prefrontal cortex (DLPFC), and this could lead to a partial dysregulation of monocytes contributing to the emergence of the syndrome. Additional important information is expected from this research line in the coming years.

## 4. Structural and Functional Changes in Tourette Patients

The neural substrate established to be pathophysiological in TS relates to habit formation. Indeed, tics are movement routines that can link sensory signals with specific motor actions, similar to what happens with habits. The neural circuit that mainly supports the generation of habits and tics connects the cerebral cortex to subcortical structures and these back to the cortex, forming multiple neural loops [42]. This concerns regions specifically involved in the pyramidal and extrapyramidal control of movement as the BG and related nuclei, the thalamus, and the brain cortices, and for this reason, it is known as the cortical-striatal-thalamus-cortical (CSTC) circuit [43].

### 4.1. Cortical-Striatal-Thalamus-Cortical Circuit and Basal Ganglia

**Figure 1:**
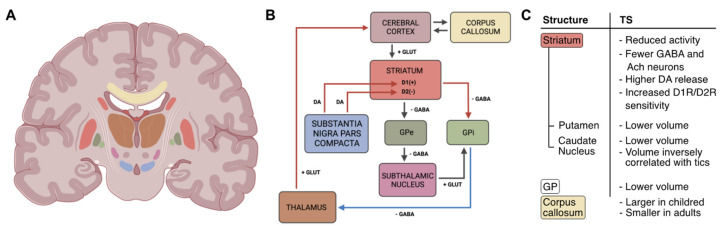
Graphical representation of the cortical-striatal-thalamus-cortical (CSTC) circuit and its changes in patients with TS. (**A**) Illustration showing the location of the basal ganglia (BG) and areas involved in the CSTC. (**B**) Scheme of the CSTC circuit: boxes’ colors indicate the brain area in (**A**); arrows represent neurotransmitter pathways (DA: dopamine; GLUT: glutamate; GABA: γ-aminobutyric acid); red/blue arrows indicate increased/reduced activity; lightning symbol refers to hyperactivity of the striatum. (**C**) Summary of the morphological and functional changes involving CSTC structures found in Tourette Syndrome (TS) patients. GP: globus pallidus; GPe: external globus pallidus; GPi: internal globus pallidus; D1(+)/D1R: dopamine 1 receptor; D2(−)/D2R: dopamine 2 receptor. The image of the brain section was created in BioRender.com.

The CSTC circuit is composed of multiple and sometimes overlapping neural pathways (see Figure 1), and tics seem to result from dysfunctions in regions that are part of this circuit [44]. In the 20th century, the clinical experience led to evidence that lesions of the putamen, globus pallidus, and subthalamic nucleus, all structures that are part of the BG, were linked with Parkinsonian signs, dystonia, and hemiballismus, which are all movement disorders [43]. The first region involved in this circuit is the cortex, which is the part of the brain assigned to the higher functions (i.e., cognitive and executive functions, such as memory, decision-making, language, or complex elaboration of sensory stimuli). The BG are targets of frontal, prefrontal, and parietal cortices, and through the lateral ventral nucleus, they, in turn, communicate with Brodmann’s area 6 of the motor cortex. This group of gray matter nuclei is densely interconnected with the thalamus, the brainstem, and the substantia nigra. Several cortical regions project to the striatum (caudate nucleus and putamen) in a limited area of the latter. The main function of this loop is the selection and initiation of voluntary movements [45]. From the striatum, the projections converge to the external (GPe) and internal (GPi) portions of the globus pallidus (GP), the latter portion projecting into specific regions of the thalamus (ventral-anterior and ventral-lateral) [46]. Functional imaging studies have shown the implication of basal ganglia (BG) in the pathophysiology of TS [18]. In most instances, a reduction in the activity of these regions was detected, including the striatum; this clearly supports a compromission in extrapyramidal motor control, but the specific mechanisms remain debated. As a matter of fact, brain imaging techniques, by analyzing brain metabolism or blood oxygenation, cannot provide us with specific information about the type of cellular and subcellular components that subtend these changes (e.g., excitatory or inhibitory). Importantly, a reduction in the number of GABA-ergic interneurons in the striatum of TS patients [47,48,49] and the effect of GABA antagonists in rodent and primate animal models of tics both suggest that disinhibition of output striatal neurons might underlie tics generation. Nevertheless, relating such findings with the resting state and higher scale observations of brain imaging studies remains challenging, also due to the complex BG circuitry. TS appears to be associated with a specific cerebral network characterized by a reduction in the activity of the BG and thalamocortical circuits [50]. A cortical-striatum-pallidus-cortical semi-open loop plastic model has been proposed, in which the BG use reinforcing signals and local competitive learning to reduce sparse cortical information [51]. As already stated, the role of the BG is to integrate various inputs coming from the cortex and use this information to select different motor and/or cognitive programs to be sent back to the thalamus and the cerebral cortex. As shown in Figure 1, the access point to the BG is the striatum (which, together with the subthalamic nucleus, is the major input structure of the BG), which receives converging information from the limbic and associative cortices. The striatum sends its GABAergic projections to the globus pallidus (the major output nucleus, which in turn sends inhibitory outputs to the thalamus) through two pathways: a direct one, which involves the GPi, and an indirect one, involving also the GPe and the subthalamic nuclei. The return to the cortex occurs through a thalamic relay. Furthermore, the cortex sends inputs directly to the subthalamic nuclei and to the external and internal GP. The direct pathway facilitates the activation of programs at the cortical level, through the inhibition of the GPi by the striatum, which usually acts as a thalamic inhibitor through its GABAergic connections. With the activation of the direct pathway, the thalamus is therefore free to send input to the cortex, initiating the movement. The indirect pathway, on the other hand, blocks the activation of the thalamic relay by increasing the activity of the GPi and thus inhibiting movements [52]. Neuroimaging studies showed morphological alterations in different BG nuclei of TS patients, especially at portions of the caudate nucleus of the striatum [53,54]. TS patients show a lower lenticular nucleus (i.e., the globus pallidus and the putamen) volume that could be a marker of TS persistence after adolescence [54]. In addition, volumes of the caudate nucleus significantly and inversely correlate with the severity of tics and OCD symptoms in early adulthood, when severity becomes stable after the usual waxing and waning [55]. Right caudate nuclei volume is lower in subjects with severe TS symptoms, and this difference subsists even with monozygotic twins [56]. Lower volume of caudate nucleus may be a long-lasting trait brain morphologic abnormality that could have non-genetic origins, at least in part. According to some studies, cortico-subcortical connections between several areas in the brain are involved in TS [53]. In particular, the frontal lobe region is the source section of thalamus-cortical targets of five functionally and structurally distinct neuronal circuits: motor circuitry, oculomotor circuitry, limbic system, dorsolateral prefrontal circuitry, and lateral frontal orbit [57]. The variety of these circuits probably explains the variety of TS subtypes and the involvement of different neurotransmitters (dopamine, serotonin, etc.). Some theories trying to explain the pathophysiological origin of TS see the emergence of tics as an excitatory anomaly of the striatum that consequently would lead to a disinhibition of the cortex and motor programs [58]. There is also evidence about the implication of language areas and the left temporo-parietal-occipital area that allows us to attribute the meaning to words, as well as frontal areas responsible for grammatical and syntactic organization. Indeed, in TS patients, increased activity has been found at the level of Broca’s area [58,59]. Interestingly, children with TS have larger corpus callosum than controls (in the rostrum and splenium components), while it appears smaller in adults. Volume changes have also been found in the prefrontal cortex and orbitofrontal cortex [60]. More specifically, dorsal prefrontal regions have been found to be larger in TS subjects if compared to controls [61]. However, when considering age and sex, such an effect on PFC volume seems to decrease with age and disappears in TS adult males, while adult TS women have smaller dorsal PFC volumes [61]. This suggests that age and sex might play a role in volume changes related to TS. Related to this, BG anatomical abnormalities encountered in childhood through brain imaging studies seem to predict the severity of tics in early adulthood [55].

### 4.2. Dopamine System

As mentioned, TS seems to be characterized by an excess of dopamine or supersensitivity of postsynaptic dopaminergic receptors at the BG level, resulting in an imbalance of CSCT circuits [62]. There are two subtypes of dopamine receptors expressed in the BG circuitry, D1-like (D1 and D5) and D2-like (D2A, D2B, D3, and D4), which respectively stimulate and inhibit cyclic adenosine monophosphate (cAMP) levels [63]. The balance between indirect and direct pathways is regulated by the differential action of dopamine on neurons of the striatum, exerted by means of diffuse projections from the neurons of the substantia nigra pars compacta [64]. Dopamine release in the striatum increases the activity of the direct pathway by acting on D1 receptors and reduces the activity of the indirect pathway by acting on D2 receptors, thus ultimately promoting movement, since both direct pathway activation and indirect pathway inhibition lead to thalamus dishibitition by decreasing the activity of GPi neurons [65]. The activation of the direct pathway leads to a reduction in the inhibitory activity of the GPi and the overall promotion of movement [65]. The gene encoding for the D2 receptor has several variants (or allelic forms) [66], and there is a strong relationship between variants of this gene and impulsive, addictive, and compulsive disorders [67,68]. It is known that psychological stressors can cause dopamine release within the ventral striatum [69] and modulate dopamine-dependent plasticity in the prefrontal cortex [70]. Moreover, high cortisol level facilitates dopaminergic neuron activation [69]. It is, therefore, necessary for a critical range of dopamine reduction for optimal cognitive functioning after exposure to stressful events [71]. Psychosocial stress may increase dopamine release in the prefrontal cortex; thus, dopaminergic activity within the ventromedial prefrontal cortex is linked to subjective stress evaluation [72]. Dopamine release, both phasic and tonic, reflects an activity system of midbrain neurons deriving from BG mechanisms [73]. There appears to be a general disorganization of the dopaminergic system in the brain of patients suffering from TS as well as OCD [62], which explains the reduction of tics observed when TS patients are treated with dopamine receptor blockers such as antipsychotic drugs [74]. In conclusion, compromised neuronal activity and enhanced dopaminergic transmission in the striatum of TS patients are thought to undermine the physiological selection of motor programs and promote their activation, thus leading to tic behavior, as shown in Figure 1.

### 4.3. Cortical and Subcortical Motor Areas

**Figure 2:**
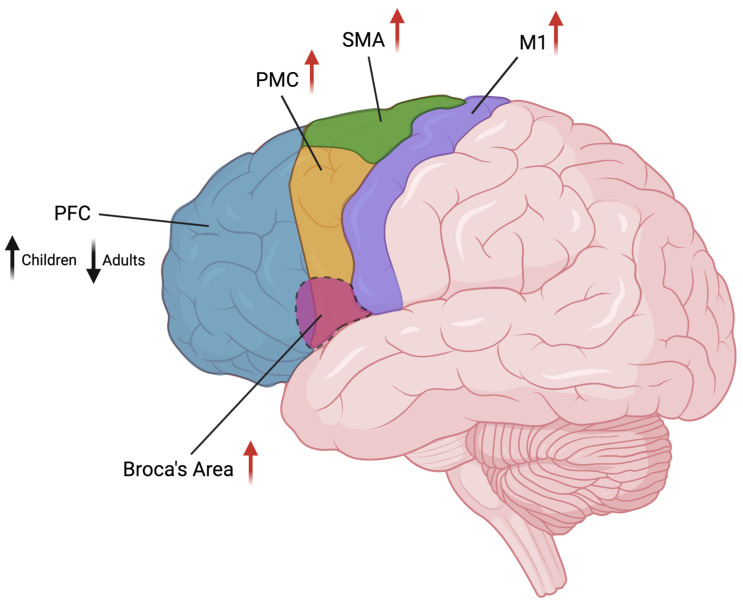
Graphical representation of the cortical areas involved in TS and their brain location. Changes in volume (black arrow) or activity (red arrows) are indicated beside area names (arrow’s direction indicates increase/decrease). PFC: prefrontal cortex; PMC: premotor cortex; SMA: supplementary motor area; M1: primary motor cortex. Image created in BioRender.com.

Previous studies provided evidence for a correlation between putamen and ventral thalamus activation, which is positive for control subjects and negative for TS patients [75]. On the other hand, a cross-circuital correlation between the supplementary motor area (SMA) and ventral striatum and between the orbitofrontal cortex and putamen are uniformly negative for controls and positive for TS patients. This could suggest that, in controls, activation of the circuit is being regulated by the primary motor cortex (M1), which directly governs motor execution, and by the lateral orbitofrontal cortex, while in TS patients, this activity is abnormally integrated and the cortical activity is mostly influenced by limbic structures [76]. Tics severity correlates with SMA, lateral premotor cortex, and lateral prefrontal cortex activity [77], an activation pattern that comprehends mainly the integration and planning areas. In these subjects, it seems that SMA overstimulation, due to an excess of unfiltered information coming from the thalamus, could be the cause of the premonitory sensation that anticipates tics (“premonitory urge”) [78]. Insula, anterior cingulate, and parietal operculum may be the regions implied in negative connotations associated with premonitory urges [79]. Furthermore, these regions are functionally connected and allow the neural representation of the body status and the initiation of behaviors associated with these representations. Motor disinhibition in TS seems also to derive from M1 excitability, which is mainly influenced by afferent inputs coming from striatum-thalamic circuits. During suppression or preparation of movement, the superior motor areas and prefrontal cortex affect M1 excitability [80]. A finger-opposition task study conducted on TS patients [81] revealed that brain connectivity, both at baseline and during the task, was altered. Using dynamic causal modeling (DCM) analysis of fMRI data collected, antagonist input forces from the premotor and right subcortical regions to the right M1 have been registered. The bigger the impact of subcortical connectivity on the M1, the more severe the motor symptoms are. The stronger the connection between the premotor area and right M1, the less severe the symptoms are. Thu, there would be two competitive forces in a tug-of-war-like mechanism: abnormal subcortical afferents to the M1, compensated by premotor cortex inputs. Those patients seem also to have higher activity of the hypothalamic–pituitary–adrenal axis, which is the main effector of individual stress response and then of the orthosympathetic noradrenergic system [82]. Furthermore, a specific metabolic pattern has been identified in TS+OCD patients. Subjects with comorbidity are characterized by reduced activity of the anterior cingulate and dorsolateral prefrontal regions, with a consequent increase in the activity of the precuneus and M1. This activation pattern is linked to symptom severity [83]. The activity of the dorsal left frontoparietal network may be linked to patients’ attempts to move their attention away from obsessive thoughts [84]. Neuroimaging studies performed on patients before and during tic occurrence also pointed out the involvement of the cerebellum in TS pathogenesis [79,85,86]. In this context, the subthalamic nucleus, which receives input from the primary motor cortex, projects to the cerebellar cortex connecting the cerebellum to the cortico-striatal-thalamo network [87]. Figure 2 summarizes the changes observed in TS patients in cortical areas and their brain location.

It seems that different urges share circuits widely independent from neural systems responsible for goal-directed voluntary action execution [88]. In TS patients, the centromedian nucleus of the thalamus seems to be affected by cingulate activation, and in turn, it influences the right insular cortex. This connectivity pattern suggests that cingulate motor region processes are involved in the selection of motor programs by the striatum through their influence on the centromedian thalamus [88]. This area could also signal the outcome of the selection process, determining if the condition that generated urges for action has been resolved and, in that case, generating the sensation of satisfaction.

In conclusion, it is worth noticing that all morphological and functional changes presented above could depend on multiple physiological and cellular factors, including changes in glial cell number, blood flow, lysosome function, alterations in the soma size, as well as in the size and number of dendritic spines without this being attributable to neural plasticity processes that involve synaptic function [89]. Nevertheless, the following paragraphs will discuss additional evidence that might support the hypothesis that functional and structural synaptic plasticity might play a role in TS-induced changes in neural organization.

## 5. Functional Plasticity Alterations in Patients with Tourette Syndrome

As described above, alteration in dopaminergic transmission in the striatum is thought to lead to aberrant firing in clusters of striatal neurons, which reflects the inhibition of GPi and substantia nigra pars reticulata (SNr). A decreased inhibitory output from BG, in turn, results in excessive frontal cortical activity, and in cortical motor areas, these disinhibited projections translate into tics [90], where the over-activation seems related to tic frequency, and long-term potentiation (LTP)-like effects to their severity [91]. Theta-burst stimulation (TBS) is a type of transcranial magnetic stimulation (TMS) that causes plasticity phenomena such as LTP and long-term depression (LTD), analyzed through changes in the amplitude of motor evoked potentials (MEPs) [92]. TBS has been applied in the two central nervous system regions probably concerned with the generation of tics (i.e., upper limb and cranial ones), M1, and the brain stem [93]. While these plastic phenomena were usually found in controls, adult subjects with TS did not show significant variation in the magnitude of MEPs after intermittent (iTBS) protocols of TBS [94,95]. Short-interval intracortical inhibition was found to be reduced in TS patients; also, brain stem excitability, analyzed through the blink reflex, was found abnormal in TS, prompting impaired excitability of brain stem interneurons [96]. The lack of the expected changes in MEP amplitudes and blink reflex late responses indicate that abnormal plasticity is involved in TS pathophysiology, as confirmed by abnormally increased cortical associative plasticity found in young patients with severe TS compared to healthy subjects [97]. At the same time, the altered LTP-like and LTD-like plasticity are probably not directly implicated in tics generation but are likely a consequence of impaired motor control [96]. Similarly, paired associative stimulation (PAS), a method involving low-frequency repeated pairs of electrical stimulation of the median nerve combined with TMS of the contralateral motor cortex [98], was not able to induce the potentiation (LTP) of MEPs, usually observed in controls, when applied to TS patients [95]. Furthermore, synaptic plasticity was found to be positively correlated with motor skills in healthy controls after 9 months. Though, TS patients did not show LTP in response to PAS and recorded lower levels of long-term motor skill consolidation [95]. Most TS patients responded with LTD-like changes, while the majority of healthy controls responded with LTP-like ones. At the same time, TS subjects with severe symptoms tended to show physiological LTP-like plasticity; less severely affected patients had LTD-like responses, suggesting an ongoing compensatory process [92]. Importantly, the successful use of behavioral therapy for the treatment of TS [99], both in children and adults, supports the idea that adaptation mechanisms such as neural plasticity may play a crucial role in the development of tic symptoms [100]. Indeed, Marsili et al. (2017) found that M1 responses to iTBS/cTBS were reduced in TS patients, indicating that synaptic plasticity processes might be anomalously recruited by tic behavior in the long term, worsening motor control and motor learning [101]. It is, therefore, clear that neural plasticity in TS brains plays a role in tics modulation and suppression. In particular, the ability to produce a plastic response assists a better control of the symptoms; its failure, in contrast, generates more severe and persistent tics in adult age. As mentioned, this plasticity could be, at the same time, the result of compensatory mechanisms implied by the motor system to reduce tics. Even transcranial direct current stimulation (tDCS) of the cerebellum has been shown to modify excitability during M1 PAS. By changing cerebellar Purkinje cell activity, tDCS could be exploited to increase LTD, preventing the over-induction of motor movements and atypical motor memories in TS [102]. Another approach is the neurosurgical method called the deep brain stimulation (DBS), which involves the implantation of electrodes in deep areas that initially proved beneficial in dystonic patients [103,104]. Later application of DBS for the stimulation of the GPi showed to be effective in a subgroup of TS patients [105,106]. The fact that plasticity seems to fail in some TS patients, leading to more severe symptoms and persistent tics, also deserves mention [107,108]. De facto, technical constraints linked to the investigation of synaptic plasticity in humans limit the currently available evidence and further experimental investigations are required to understand TS pathophysiology and bridge the gap from research to clinical application.

## 6. Striatal Synaptic Plasticity as a Possible Pathophysiological Mechanism

As presented in the previous paragraphs, both structural and functional anomalies of BG have been widely documented and are supposed to contribute to the induction and expression of TS. Focusing on the neuronal microcircuitry, one of the known manifestations in TS patients is a ~60% reduction of parvalbumin-positive (PV+) neurons in both associative and sensorimotor portions of the dorsal striatum (DS) [47,48,49]. In the DS, PV+ neurons are GABAergic fast-spiking (FS) interneurons exerting local inhibitory control over the most abundant type of striatal neuron, the medium spiny neuron (MSN) [109]. Synaptic plasticity has been widely characterized in the striatum of rats and mice both functionally and molecularly. Either LTP or LTD forms of plasticity have been demonstrated on glutamatergic afferents of the DS, both requiring the concurrent release of DA (see [110] for review). LTD in the striatum is classically induced by high-frequency stimulation (HFS) and requires D2 but not NMDA receptors activation [111]. More specific analysis using intracellular recordings has shown that LTD of excitatory synapses at MSNs requires the activation of postsynaptic metabotropic glutamate receptors (mGluRs) beside D2 receptors, as well as endocannabinoid (eCB) signaling [112,113]. This form of LTD can occur for both the direct and the indirect pathway MSNs, but differences exist in terms of the receptors involved and the origin of DA release [114]. Interestingly, dopamine-dependent LTD in the striatum has been suggested to drive the reduction in glutamate transmission efficiency naturally occurring during early postnatal development [115], while other authors showed a developmental switch from NMDA-dependent LTP to NMDA-independent LTD following HFS in the dorsolateral striatum [116]. Both phenomena might be involved in the early emergence of TS in the presence of DA signaling compromission. Albeit much less characterized, LTP in the striatum generally requires NMDA and D1 receptors activation, although with differences between the two classes of MSNs. Recent studies based on spike-time-dependent plasticity indicate that LTD or LTP can be enabled by DA acting on a silent eligibility trace initiated earlier in time [117]. FS interneurons are reached by glutamatergic cortical axons as well, and their synapses are subjected to similar LTD/LTP plasticity phenomena [109]. Hence, DA-dependent synaptic plasticity of corticostriatal excitatory projections represents a powerful mechanism for the regulation of BG functioning and, more in general, motor programs. As a matter of fact, several studies addressed this hypothesis in animal models of Parkinson’s disease (PD) [113,118], while no evidence is available about the implication of these forms of plasticity in the expression of tic behaviors. A study using optogenetic stimulation of corticostriatal synapses showed that such hyperactivation is able to induce OCD-like repetitive behaviors in mice [37], but this was dependent on low frequency and chronic stimulation and limited to the ventromedial striatum. The involvement of eCB signaling in striatal plasticity and the formation of motor habits [119] should be considered in light of current developments in experimental TS treatment based on cannabinoids. Indeed, THC and Sativex have already been approved for the treatment of spasticity and chemotherapy nausea, and these drugs are also gaining attention for treating TS symptoms. Few studies evaluated THC effects on TS tics and obsessive-compulsive behavior as well as collateral effects on cognitive functions. Albeit the results are far from conclusive, these studies found a significant reduction of tics and behavioral deficits after THC treatment, using standard evaluation scales [120].

Apart from a possible role in the etiopathology of TS and in its emergence during childhood development, striatal synaptic plasticity might also contribute to the maintenance of tic-like behaviors over time. This hypothesis has only been partially addressed using computational modeling [121]. On the other hand, it has been experimentally found that anomalous recurrent activation of BG through the cortico-basal ganglia-thalamo-cortical (CBGTC) loop can efficiently drive synaptogenesis with different consequences on direct and indirect pathways [122], possibly leading to deficits in motor control.

### Immunological Bases of Tourette Syndrome and Striatal Plasticity

In the context of striatal plasticity, immunopathogenic mediators are thought to be involved in TS development, and their role might be central. Several lines of evidence suggest that increased levels of proinflammatory cytokines, including tumor necrosis factor-α (TNF-α) and interleukin-12 (IL-12), but also autoantibodies with potential nervous system pathogenicity, are found in patients with PANDAS and related tic disorders, including TS, Sydenham’s Chorea (SC) and OCD [123,124]. Based on these data, investigators developed animal models of TS by immunizing mice for GABHS [125,126], which displayed increased repetitive behaviors, high concentrations of IgG in the brain and anti-brain antibodies, or by directly infusing patients’ sera in the striatum with mixed results, but for the most part leading to increased stereotyped movements [20,127,128,129,130]. Recent studies provided more specific results in terms of cells targeted by these autoimmune mechanisms: Xu and colleagues showed that IgGs from PANDAS pediatric patients affect the electrical activity of cholinergic interneurons in the striatum [131], further involving them in plasticity processes hypothetically linked to TS development, as these interneurons are required for LTD at corticostriatal synapses [114]. Another example is provided by the work of Yeh et al. (2012), who identified specific antibodies against hyperpolarization-activated cyclic nucleotide channel 4 (HCN4) in TS patients’ serum [132]. The effects of these anti-brain antibodies are still unknown, but HCN channels have been involved in the physiological activity of striatal cholinergic interneurons as well [133,134], thus deserving further experimental analyses. NMDA receptors, widely involved in cortico-striatal synaptic plasticity, might also be ideal targets for future investigations. As a matter of fact, autoimmune targeting of NMDA receptors has already been shown to underlie other brain pathologies, such as specific encephalitis [135], and is discussed as a potential etiopathological route for other mental disorders [136]. In a more general perspective, the role of the immune system in regulating synaptic plasticity has been established for decades [137], as well as the role of microglial cells in synaptic pruning processes, which is of crucial importance during development [138]. De facto, specific effects of immune response mediators and microglial cell activity have been evidenced in the striatum and are already implicated in other motor-related disorders (see [139] for an interesting review on this topic).

## 7. Conclusions

In this review, we summarized the current knowledge about the anatomical and functional brain changes that have been involved in the expression of TS tics. In addition, we described specific anomalies in functional cortical plasticity that have been observed in TS patients using neuromodulation. Finally, we introduced the established evidence about the plastic potential of BG microcircuits and discussed possible implications in the development and maintenance of TS. Importantly, an increasing number of studies are testing the effectiveness of noninvasive brain stimulation interventions aimed at ameliorating the TS symptomatology to improve the quality of life of this clinical population. However, the lack of comprehensive knowledge about the pathophysiological mechanisms of TS could undermine clinical trial results. Hence, further studies are required to unveil the link between neural plasticity phenomena, including synaptic plasticity, and TS development. It is important to emphasize again, as we did above, the fact that the investigation of synaptic plasticity in humans is still very limited by the available tools, which still hinders a deeper understanding of TS pathophysiology. A more comprehensive view would allow us to explain alterations in cortical and subcortical connectivity observed in TS, as well as the other manifestations of the syndrome, including dysregulated cognitive control [140] and comorbidity with OCD and ADHD [47,141,142], also paving the way for more focused and effective therapeutic interventions.

## Data Availability

No data were generated for this study.
